# Coeliac Disease Case–Control Study: Has the Time Come to Explore beyond Patients at Risk?

**DOI:** 10.3390/nu15051267

**Published:** 2023-03-03

**Authors:** Gemma Castillejo, Carlos Ochoa-Sangrador, David Pérez-Solís, Maria Luz Cilleruelo, Ester Donat, Jose Ignacio García-Burriel, Félix Sánchez-Valverde, Salvador Garcia-Calatayud, Francisco Javier Eizaguirre, Eva Martinez-Ojinaga, Patricia Barros, Rosaura Leis, Jose Carlos Salazar, Josefa Barrio, Luis Peña-Quintana, Verónica Luque, Isabel Polanco, Carmen Ribes, Enriqueta Roman

**Affiliations:** 1 Unitat de Recerca en Pediatria, Hospital Universitari Sant Joan de Reus, Nutrició i Desenvolupament Humà, IISPV, Universitat Rovira i Virgili, 43204 Reus, Spain; 2 Complejo Asistencial de Zamora, 49022 Zamora, Spain; 3 Pediatrics, Hospital Universitario San Agustín, 33401 Avilés, Spain; 4 Hospital Universitario Puerta de Hierro, Majadahonda, 28222 Madrid, Spain; 5 Pediatric Gastroenterology and Hepatology Unit, Celiac Disease and Digestive Immunopathology Unit, Instituto de Investigación Sanitaria La Fe, Hospital Universitari i Politècnic La Fe, 46026 Valencia, Spain; 6 Complejo Hospitalario Universitario de Vigo, 36312 Vigo, Spain; 7 Navarre Children’s Nutrition and Digestive Study Group (GENDINA), Department of Paediatrics, NAVARRA BIOMED, University Hospital of Navarra, 31008 Pamplona, Spain; 8 Hospital Universitario Marques de Valdecilla, 39008 Santander, Spain; 9 Paediatric Gastroenterology, Hepatology and Nutrition Unit, Hospital Universitario Donostia, 20006 San Sebastian, Spain; 10 Department of Pediatric Gastroenterology, Hospital Universitario La Paz, 28046 Madrid, Spain; 11 Hospital San Pedro de Alcántara, 10071 Cáceres, Spain; 12 Unit of Pediatrics Gastroenterology, Hepatology and Nutrition, Pediatrics Department, Hospital Clínico Universitario de Santiago-USC, IDIS, CiberObn, 15706 Santiago de Compostela, Spain; 13 Hospital Universitario Infantil Virgen del Rocío, 41013 Sevilla, Spain; 14 Hospital Universitario de Fuenlabrada, Fuenlabrada, 28942 Madrid, Spain; 15 Pediatric Gastroenterology, Hepatology and Nutrition Unit, Complejo Hospitalario Universitario Insular Materno-Infantil, CIBEROBN ISCIII-Universidad de Las Palmas de Gran Canaria, 35016 Gran Canaria, Spain; 16 Unitat de Recerca en Pediatria, Nutrició i Desenvolupament Humà, Universitat Rovira i Virgili, 43007 Tarragona, Spain

**Keywords:** coeliac disease, pediatric gastroenterology, screening, general practice

## Abstract

The worldwide prevalence of asymptomatic coeliac disease (CD) is increasing, which is in part due to the routine screening of children with risk factors. Both symptomatic and asymptomatic patients with CD are at risk of long-term complications. The objective of this study was to compare the clinical characteristics of asymptomatic and symptomatic children at the time of CD diagnosis. A case–control study was conducted using data from a cohort of 4838 CD patients recruited from 73 centers across Spain between 2011 and 2017. A total of 468 asymptomatic patients (cases) were selected and matched by age and sex with 468 symptomatic patients (controls). Clinical data, including any reported symptoms, as well as serologic, genetic, and histopathologic data were collected. No significant differences were found between the two groups in most clinical variables, nor in the degree of intestinal lesion. However, the asymptomatic patients were taller (height z-score −0.12 (1.06) vs. −0.45 (1.19), *p* < 0.001) and were less likely to have anti transglutaminase IgA antibodies ≥ 10 times the upper normal limit (66.2% vs. 758.4%, *p* = 0.002). Among the 37.1% of asymptomatic patients who were not screened for CD due to the absence of risk factors, only 34% were truly asymptomatic, while the remaining 66% reported non-specific CD-related symptoms. Therefore, expanding CD screening to any child who undergoes a blood test could reduce the burden of care for some children, as many of those considered asymptomatic reported non-specific CD-related symptoms.

## 1. Introduction

Coeliac disease (CD) is an immune-mediated systemic disorder triggered by gluten and related prolamins in genetically susceptible individuals. It is characterized by a variable combination of gluten-dependent clinical manifestations, CD-specific antibodies, HLA-DQ2/8 haplotypes, and enteropathy [1]. Over the past few decades, the presentation of CD has changed worldwide, with the diagnosis occurring at an older age [2] and a greater number of patients being diagnosed with more subtle symptoms or even as asymptomatic [3]. This change is partly due to the recognition that the disease can occur at any age and the introduction of screening in high-risk children, who are often asymptomatic. Moreover, the asymptomatic CD is becoming more common, as shown by some studies in which almost 1% of asymptomatic healthy adolescents screened (without risk factors for CD) had CD [4]. Screening is recommended for first-degree relatives of CD patients and in children with a concomitant disease (chromosomal abnormalities such as Downs, Turner, or Williams syndrome, as well as patients with another autoimmune disease, such as type 1 diabetes, autoimmune thyroid, or autoimmune hepatitis, among others), as CD patients have a 3- to 10-fold higher risk of developing another autoimmune disease [5,6,7,8]. It is worth noting that a recent US mass screening study performed in 9973 children found that 90% of asymptomatic positive children did not have a first-degree relative affected with CD [9]. This highlights the importance of widespread screening for CD, as relying solely on symptoms or risk factors such as family history may miss many cases. Furthermore, it is important to note that other studies have found that the serological, histological, and genetic characteristics of symptomatic and asymptomatic CD patients are similar [10,11]. Given the high prevalence of this disease [12], particularly in Europe, and the potential for serious complications if left untreated [13], it is crucial to investigate the characteristics of patients with or without symptoms at the time of diagnosis. This information can be used to inform decision-making in the healthcare practice. The aim of this study is to compare the characteristics of asymptomatic and symptomatic CD patients at the time of diagnosis.

## 2. Materials and Methods

### 2.1. Study Design and Population

The present study was designed as a case–control study, embedded in the REPAC2 cohort [14]. This was a nationwide, prospective, observational, multicenter registry of new CD cases recorded between January 2011 and June 2017. The CD Working Group of the Spanish Gastroenterology, Hepatology, and Pediatric Nutrition Society (SEGHNP) invited all pediatric gastroenterology departments in Spain to participate in the study. Of the 117 pediatric gastroenterology units in Spain, 73 (62.4%) from 15 of the 17 Spanish regions agreed to participate. The inclusion criteria for this study were patients under 15 years of age who were diagnosed with CD at participating centers after the study start date. To be included in the cohort, patients had to meet the diagnostic criteria established by the European Society for Pediatric Gastroenterology Hepatology and Nutrition (ESPGHAN) at the time of diagnosis. For patients diagnosed before 2011, the 1990 ESPGHAN criteria were used [15], while for those diagnosed since 2011, the 2012 criteria were used [16]. To clarify, before 2011, all patients diagnosed with CD had to undergo an intestinal biopsy for diagnosis. However, since 2012, patients can be diagnosed without undergoing an intestinal biopsy, if they meet the diagnostic criteria established by the ESPGHAN. Asymptomatic CD cases and a group of symptomatic controls were selected for the study, with each case being matched 1:1 by center, age, and sex. Children were considered asymptomatic if they did not have any CD-suggestive symptoms according to the ESPGHAN diagnostic guidelines [1,16]. However, some of the so-called asymptomatic children had other complaints that were not considered in the guidelines and were thus categorized as having “non-specific CD symptoms” for the purposes of the study.

### 2.2. Data Analysis and Management

The data for this study were collected through an electronic questionnaire hosted on the SEGHNP website. The questionnaire collected information on various aspects of the patient’s medical history, including demographics, mode of delivery, breastfeeding history, CD family history, CD-specific symptoms, height and weight, associated conditions, serology, biopsy, and genetic results. Clinical presentations were classified as either asymptomatic or symptomatic based on the presence or absence of symptoms established by the ESPGHAN diagnostic guidelines [1,16]. Symptomatic children had at least one of the signs or symptoms mentioned in the guidelines: chronic or intermittent abdominal pain, diarrhea, constipation or bloating, distended abdomen, recurrent nausea and/or vomiting, tiredness and lethargy, weight loss, failure-to-thrive, stunted growth/short stature, delayed puberty, amenorrhea, irritability, chronic fatigue, neuropathy, arthritis/arthralgia, chronic iron-deficiency anemia, decreased bone mineralization, recurrent aphthous stomatitis, dermatitis herpetiformis, dental enamel defects, and abnormal liver biochemistry. Asymptomatic patients were evaluated to determine if they belonged to a risk group, such as first-degree relatives of patients with celiac disease, those who already had another autoimmune disease, and those with chromosomal diseases, to see if a blood test for CD detection was indicated. The study team collected information on why the blood test was performed for asymptomatic patients who did not belong to a high-risk group. The responses were collected in an open text box and analyzed to determine the reason for the blood test. If data were missing, patients were not excluded, and no imputation was performed. To summarize, asymptomatic patients were classified into three groups: the first group included patients with high-risk factors such as first-degree relatives of CD, and those with other autoimmune or chromosomal diseases. The second group included asymptomatic children who had symptoms not listed in the guidelines (non-specific CD symptoms), and the third group included patients with no symptoms at all.

To calculate the z-scores for height, weight, and body mass index (BMI), the study used the growth references and standards provided by the World Health Organization (WHO) [17,18]. A z-score of 0 indicates that a child’s measurement is equal to the median, while a z-score of +1 or −1 indicates that the measurement is one standard deviation above or below the median, respectively.

Serology tests for celiac disease were performed at each participating center to detect anti transglutaminase IgA antibodies (TGA-IgA) and anti endomysial IgA antibodies (EMA-IgA) based on the available methods in their laboratories, with specific cut-off values determined for each method. The antibody titer for each patient was recorded, along with the cut-off value used. HLA typing was also performed locally, and patients were grouped as DQ2 or DQ8 based on the results. Some centers performed complete genotyping, while others determined only DQ2/8 positivity or negativity. All patients underwent fibrogastroduodenoscopy, which was performed under sedation, and macroscopic and microscopic findings were recorded. The biopsy samples were evaluated by the pathologist at each center and classified according to the Marsh–Oberhuber classification. [19]. In this study, samples classified as Marsh 2 to 3 according to the Marsh–Oberhuber classification were considered indicative of CD [16].

### 2.3. Statistical Analysis

The study was designed with an expected sample size of 468 cases and 468 controls, with a power of 86.8% and an alpha error of 0.05 to estimate differences of more than 10% between groups. The distribution of continuous variables was assessed using histograms and Kolmogorov–Smirnov test. Continuous variables were reported as mean and standard deviation or median and interquartile range (IQR) based on their distribution. Chi-squared tests were used for group comparisons for categorical variables, while Student’s *t*-test and ANOVA or Mann–Whitney U-tests and Kruskal–Wallis test were used for group comparisons for continuous variables, depending on their distribution. Statistical significance was accepted at *p* < 0.05, and IBM SPSS Statistics version 26 (IBM Corp., Armonk, NY, USA) was used for all statistical analyses.

### 2.4. Ethics and Approvals

The investigations were carried out according to the principles of the Declaration of Helsinki. Informed consent was obtained from the parents or legal guardians of the participants. This study was approved by the Research Ethics Committee of the Hospital Universitario Puerta de Hierro, Majadahonda, Madrid (263.2011), and individually, by all participating centers.

## 3. Results

The study included a total of 4838 celiac children, and out of these, 468 were identified as asymptomatic (9.67% of the total population). The mean age of the asymptomatic patients was 7.8 years, with an interquartile range of 4.7–11.2 years. These 468 asymptomatic coeliac patients (cases) were then matched with 468 symptomatic coeliac patients to serve as controls for the study.

During randomization, we selected 97 non-biopsy controls who were diagnosed after 2012 and met the criteria for using that approach. Since we were unsure whether there were differences between asymptomatic and symptomatic patients depending on whether they underwent a biopsy, we performed duplicate analyses. One set of the analyses included the controls randomly selected, which included 97 cases without a biopsy, while the other set only selected the controls who were diagnosed with a biopsy. However, no significant differences were found between the two analyses. Therefore, we used the randomly selected cases for the analyses, and the 97 children diagnosed without a biopsy have missing values of the Marsh lesion grade.

Table 1 shows the comparative characteristics in cases and controls.

**Figure 1:**
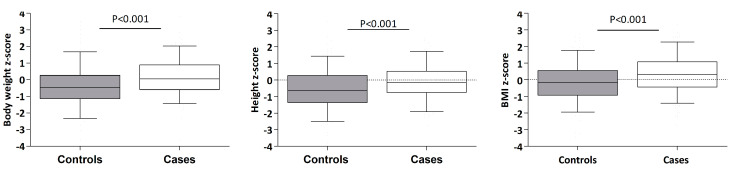
Anthropometrical measurements of cases and controls at diagnosis.

No significant differences were found between the two groups in terms of mode of delivery, breastfeeding, rotavirus vaccination, age at gluten introduction, anti endomysial IgA antibody positivity, or HLA type. The asymptomatic group exhibited a higher prevalence of risk factors, including autoimmune diseases such as type 1 diabetes mellitus and thyroiditis, Downs syndrome, and a first-degree family member with celiac disease compared to the symptomatic group. Moreover, asymptomatic children had slightly higher weight and height measurements than the symptomatic children (Figure 1).

Notably, the height of symptomatic children was below the mean for the general population, while in the asymptomatic cases, it was distributed around the normal values of the population.

**Figure 2:**
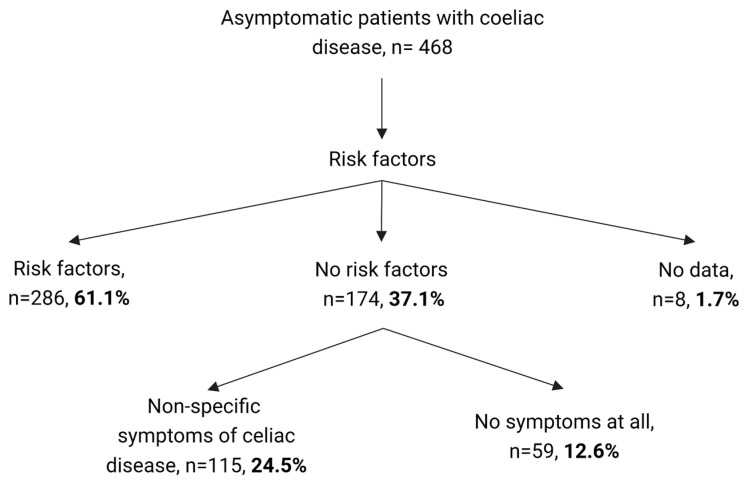
Categorization of cases.

Compared to symptomatic patients, asymptomatic patients were less likely to have a TGA-IgA value ≥ 10 times the upper limit of normal (ULN) (66% vs. 75%, *p* = 0.002) and had a higher proportion of milder lesions (Marsh 2 and 3a), while the symptomatic controls had a greater proportion of Marsh 3b and 3c. A review of the reasons behind conducting TGA-IgA tests on asymptomatic patients demonstrated that a significant proportion (up to 61.1%) of them belonged to high-risk groups identified by the ESPGHAN diagnostic protocol, such as individuals with genetic syndromes, first-degree relatives of CD patients, or other autoimmune diseases. However, 37.1% of the tests were performed without following the protocol, with some patients exhibiting non-specific symptoms associated with CD (24.5%), while others were completely asymptomatic (12.6%) (Figure 2).

Upon further investigation, recruiters reached out to families to inquire about the reason for conducting TGA-IgA tests on asymptomatic children. Of the 37.1% of patients without risk factors, 12.6% underwent testing as part of non-urgent preoperative checks, evaluations for familial hypercholesterolemia, or routine assessments upon starting care with a new pediatrician or family doctor. The remaining 24.5% had a medical concern unrelated to CD, such as allergy evaluations for recurring wheezing, food or respiratory allergies, atopic dermatitis, or urticaria, as well as assessments for non-specific skin lesions such as eczema or lichen, chest pain, halitosis, dizziness, recurrent URTIs, polyphagia, syncope, or dysphagia.

Table 2 shows the characteristics of all asymptomatic cases according to the reason for screening.

Among the asymptomatic cases, the only significant difference found was related to the age of gluten introduction, with those with a family history of CD introducing gluten at a later age compared to those without such a history (months; *p* = 0.022). No other significant differences were found.

## 4. Discussion

To the best of our knowledge, this case–control study represents the largest sample of asymptomatic coeliac patients that have been characterized to date. The findings in this study suggest that the diagnosis of CD in asymptomatic patients is not limited to high-risk groups, and this detailed analysis indicates that asymptomatic CD patients, regardless of their risk group status, share similar characteristics. The study revealed that although celiac disease may present as milder in asymptomatic cases (with lower TGA-IgA levels and less severe intestinal damage), these variations do not have any significant impact on the diagnosis. As we have seen in the results of this study, families with celiac relatives may delay introducing gluten to their children’s diets due to concerns about the potential risk of developing celiac disease. Further, it has been described that undiagnosed CD can have significant impacts on one’s physical and general development, leading to long-term complications [10,20], such as anemia, dental enamel defects [21], osteoporosis [22,23], or underachievement [24,25], which can remain permanent if CD is not treated [13].

The observations of the CD patients in the studied population align with previous research findings, indicating that height impairment in children can occur before the onset of symptoms [26,27]. The potential for irreversible growth impairment in the pediatric age group makes early detection and treatment of CD crucial. It has been described that almost half of the asymptomatic patients have minor symptoms that may go unnoticed until they improve after initiating the gluten-free diet (GFD) [10,28,29,30]. A Dutch study conducted some years ago proposed that patients be given the option to decide for themselves whether to adhere to a GFD, as some asymptomatic individuals who initially declined the diet may choose to begin it early if symptoms eventually worsen [29].

The proportion of asymptomatic patients in the studied population was 9.67%, which is consistent with other international cohorts such as those from Finland [10], the UK [31], Saudi Arabia [32], the Netherlands [33], and New Zealand [34]. However, a central European cohort showed a higher percentage of asymptomatic cases, ranging from 12.1% in Croatia to 26.5% in Italy [35]. The reason for specifically studying asymptomatic patients in this cohort is due to the increasing worldwide prevalence of asymptomatic cases, as mentioned earlier [3]. An indication of the increasing importance of this group of asymptomatic patients is reflected in the fact that, for the first time, the revised ESPGHAN criteria published in 2020 allow for diagnosis without biopsy in asymptomatic patients, the majority of whom belong to high-risk groups, as was significantly observed in this cohort. However, as results from this study and others [9] have shown, it may not be sufficient to limit CD screening to at-risk groups. In the present study, over a third of asymptomatic children were diagnosed due to TGA-IgA being included as a basic parameter in blood tests, even though this is not indicated by diagnosis guidelines. While this determination is justified in asymptomatic patients from risk groups due to the increase in prevalence, we have observed that the other two groups of asymptomatic patients behave the same. This leads the authors to question whether the inclusion of TGA-IgA plus total IgA as part of the basic biochemical profile may be justified in children whose pediatrician or family practitioner decides to carry out a blood test. This approach could reduce the gap between diagnosed and undiagnosed patients in both children and adults, which the literature has placed at between 75 and 90% over the years [26,36,37,38]. Bringing the diagnosis forward to the pediatric age could also help avoid the overuse of healthcare services and medication that have been described prior to CD diagnosis in both pediatric and adult age groups [39,40,41,42,43], as well as the delay in diagnosis in adulthood, which has been reported to be up to 10 years [44], despite the presence of CD-suggestive symptoms. The authors would like to emphasize that while CD screening is mandatory in at-risk children and meets most of the screening criteria set by the World Health Organization, universal screening for the general population, at least in adults, is currently controversial [45], although it seems to be cost-effective [43,46]. In the meantime, an opportunistic approach to CD screening could be considered for non-at-risk children who undergo blood testing for unrelated conditions. Furthermore, other recent studies have also suggested broader screening beyond just at-risk groups [9,47]. Blood testing is not common in pediatric practice and is usually performed for health or developmental reasons.

However, two important points should be emphasized. First, our study found that asymptomatic patients are less likely to have TGA-IgA levels above 10 times the UNL, which implies that they are more likely to require a biopsy to confirm the diagnosis. Second, some patients with potential CD are asymptomatic, and therefore, transient low positive serology results may be found [48]. Therefore, CD diagnosis in children, especially those with low–intermediate TGA values, should be performed in centers with sufficient experience and a carefully validated laboratory method using appropriate tests. If a child’s blood test in a general pediatric practice shows a TGA-IgA level above the threshold, the child should be referred to a specialized center for a definitive diagnosis before starting a GFD, since the misdiagnosis of CD can have long-lasting consequences. Up to 55% of children with potential CD may never develop overt CD [49,50,51,52].

The authors have identified some reasons for not performing TGA-IgA testing in asymptomatic individuals who are not part of high-risk populations. One reason may be the assumption that these patients would have lower compliance with the GFD and that asymptomatic individuals would not benefit from the GFD. However, little evidence suggests otherwise [28], and in general, compliance with a GFD among asymptomatic CD patients is generally good, particularly in children [28,53]. In some cases, compliance can be worse, as has been observed in a small group of patients [54]. In addition, the assumption that asymptomatic individuals would not benefit from the GFD may not be entirely accurate. Only 12.8% of the patients in this cohort were truly asymptomatic (Figure 2), and other studies have reported similar results, with many asymptomatic patients presenting with minor symptoms that improve after starting the GFD [10,28,30]. Concerns about the potential negative impact of starting a lifelong GFD without symptoms may also be unfounded. Recent studies comparing the quality of life and dietary adherence of children diagnosed through screening or symptoms have shown no difference between the two groups [29,53]. Instead, some authors suggest a different approach to the treatment and follow-up of this patient group to avoid decreased quality of life or increased anxiety [55]. Allowing patients to make their own decision may also be appropriate, as some initially asymptomatic patients who declined the diet may later choose to start it as their symptoms worsen [29].

Based on the mean prevalence of 0.74% [56] for undiagnosed CD/asymptomatic patients (with a range of 0.10–3.03%), including TGA-IgA as part of the basic analysis profile in children undergoing blood testing can allow the diagnosis of up to 280/100,000 children without risk factors, considering that 37.1% of the asymptomatic children in the authors’ study were diagnosed with CD. Therefore, the proposed “diagnostic approach of opportunity” could be more cost-effective than screening the general population.

Additional studies are needed to evaluate the actual cost-effectiveness of this approach, its generalizability, and whether follow-up for these patients should be designed differently to identify any issues arising from adherence to a strict diet.

## 5. Conclusions

Currently, there are still many undiagnosed CD patients. Screening has been implemented in children belonging to risk groups for CD, such as first-degree relatives or those affected by another autoimmune or chromosomal disease. Asymptomatic cases are usually diagnosed in these at-risk populations. This study’s findings have allowed for the verification of symptomatic and asymptomatic patients being identical in a large cohort; the only difference is that the asymptomatic patients present less severe intestinal damage and less impact on their nutritional state, which is beneficial in the pediatric age since they are in a period of growth.

Considering the fact that other studies have shown that complications are possible even in asymptomatic patients, but universal screening in the general population remains controversial, we propose an opportunistic screening approach beyond children at risk of CD. Therefore, we suggest exploring the existence of CD in non-at-risk children who undergo a blood test for an unrelated condition. Blood testing is not that common in pediatric practice, and when it is performed, it is usually for health or developmental reasons. By adopting this opportunistic approach, the burden of care and the risk of possible long-term complications in asymptomatic cases would be reduced. Further studies are needed to determine the cost-effectiveness of this approach and whether follow-up care should be designed differently to identify problems arising from adherence to a strict diet.

## Figures and Tables

**Table 1:** Characteristics of asymptomatic and symptomatic patients at diagnosis.

	Cases (*n* = 468) Asymptomatic at Diagnosis	Controls (*n* = 468) Symptomatic at Diagnosis	*p*-Value
Background information			
Male sex	199 (42.5%)	199 (42.5%)	NS
Age (years)	8.2 (5.3–11.4)	7.8 (4.8–11.2)	NS
Delivery type (C section)	87 (18.6)	107 (22.9)	NS
Breastfeeding	103 (76.3)	87 (79.2)	NS
Rotavirus vaccination	69 (14.7)	73 (15.6)	NS
Age at gluten introduction (months)	6 (6–8)	6.5 (6–8)	NS
Weight (z-score)	0.16 (1.06)	−0.45 (1.19)	<0.001
Height (z-score)	−0.12 (1.06)	−0.54 (1.22)	<0.001
BMI (z-score)	0.32 (1.15)	−0.19 (1.19)	<0.001
Risk factors			
Relatives with CD			<0.001
No	250 (54.1%)	383 (82.7%)	
First degree	161 (34.8%)	29 (6.3%)	
Second degree	42 (9.1%)	47 (10.2%)	
First and second degree	9 (1.9%)	4 (0.9%)	
Diabetes	62 (13.4%)	4 (0.9%)	<0.001
Thyroiditis	18 (3.9%)	5 (1.1%)	0.006
Down Syndrome	10 (2.2%)	0 (0.0%)	0.001
Laboratory parameters			
IgA antiendomysium +	325 (97.3) ^a^	328 (98.2) ^a^	NS
TGA-IgA ≥ 10xUNL	310 (66.2)	353 (75.4)	0.002
HLA	(*n* = 409)	(*n* = 393)	NS
DQ2	345 (84.4%)	338 (86.0%)	
DQ2/DQ8	40 (9.8%)	34 (8.7%)	
DQ8	20 (4.9%)	11 (2.8%)	
Others	4 (1.0%)	10 (2.5%)	
Marsh lesion grade	(*n* = 468)	(*n* = 371)	0.017 ^b^
2	29 (6.2%)	17 (4.6%)	
3a	162 (34.6%)	96 (25.9%)	0.002 ^c^
3b	188 (40.2%)	181 (48.8%)	
3c	89 (19.0%)	77 (20.8%)	

N (%) or mean (SD) or median (interquartile range). ^a^ Only available in 334. ^b^ Chi2 linear trend. ^c^ Post hoc analysis. Marsh 2 or 3a versus 3b or 3c. BMI: body mass index; CD: coeliac disease; UNL: upper normal limit; and TGA: anti transglutaminase IgA antibodies.

**Table 2:** Asymptomatic cases according to the reason for screening *.

	CD Risk Factors ^a^ *n* = 286	Non-Specific CD Symptoms ^b^ *n* = 115	Other Reasons *n* = 59	*p*-Value
Background information				
Male sex (% from total)	126 (44.1%)	45 (39.1%)	23 (39.0%)	0.577
Age (years)	8.3 (5.3–11.4)	8.1 (4.7–11.2)	7.6 (4.4–11.5)	0.603
Age at gluten introduction (months)	7 (6–8)	6 (6–7)	6 (6–7)	0.022
Weight (z-score)	0.15 (0.97)	0.14 (1.30)	0.12 (0.88)	0.973
Height (z-score)	−0.16 (1.02)	−0.07 (1.21)	−0.16 (0.89)	0.716
BMI (z-score)	0.35 (1.03)	0.24 (1.41)	0.29 (1.15)	0.686
Laboratory parameters	*n* (% from total)			
IgA antiendomysium +	196 (96.5%)	91 (98.9%)	30 (96.7%)	0.511
TGA-IgA ≥ 10xUNL	192 (67.1%)	74 (64.3%)	40 (67.8%)	0.550
HLA				NS
DQ2	205 (82.3%)	85 (84.2%)	48 (92.3%)	
DQ2/DQ8	29 (11.6%)	8 (7.9%)	3 (5.8%)	
DQ8	12 (4.8%)	7 (6.9%)	1 (1.9%)	
Others	3 (1.2%)	1 (1.0%)	0 (0.0%)	
Marsh lesion grade				NS
2	13 (4.5%)	11 (9.6%)	5 (8.5%)	
3a	108 (37.8%)	31 (27.0%)	19 (32.2%)	
3b	104 (36.4%)	50 (43.5%)	31 (52.5%)	
3c	61 (21.3%)	23 (20.0%)	4 (6.8%)	

* For 8 patients, no reason for screening was noted in the open box text N (%) or mean (SD) or median (interquartile range). ^a^ Family history of CD: 186, endocrine diseases: 82 (diabetes and hypothyroidism), genetic disorders: 12 (Downs syndrome; Williams syndrome), and rheumatic diseases: 6 (juvenile idiopathic arthritis) ^b^ Non-specific CD symptoms, such as allergy studies, non-specific skin lesions, halitosis, dizziness, and recurrent URIs, among others.

## Data Availability

Data are available on request.

## Supplementary Materials

The following supporting information can be downloaded at: https://www.mdpi.com/article/10.3390/nu15051267/s1. File S1: Complete list of authors.
