# Supplementary material for: Coeliac Disease Case–Control Study: Has the Time Come to Explore beyond Patients at Risk?
Source: Nutrients. 2023 Mar 3;15(5):1267. doi: 10.3390/nu15051267 (PMC10005316; doi:10.3390/nu15051267)
Supplement: Supplementary file 1 [file nutrients-15-01267-s001.zip › nutrients-2234316-supplementary.pdf]

Supplementary online. List of collaborators.

In addition to those listed as authors, the following members of the SEGHNP Coeliac Disease Working Group participated as recruiters for the REPAC2 Spanish National Registry of Paediatric Coeliac Disease.

Vicente Varea. Hospital Sant Joan de Deu. Barcelona, Spain.

Honorio Armas. Hospital Universitario de Canarias. Tenerife, Spain.

Rosa Solanguren. Complejo Universitario de Toledo. Toledo, Spain.

Ruth García. Hospital Materno Infantil Miguel Servet. Zaragoza, Spain.

Luis Ortigosa. Hospital Universitario Virgen de la Candelaria. Tenerife, Spain.

Pedro Urruzuno. Hospital Universitario Doce de Octubre. Madrid, Spain.

Pilar Codoñer. Hospital Universitario Dr. Peset. Valencia, Spain.

Maria Lluïsa Masiques. Hospital General de Granollers. Barcelona, Spain.

Zuriñe Garcia. Hospital Universitario de Araba-Txangorritxu, Spain.

Gonzalo Galicia. Hospital Universitario de Guadalajara. Guadalajara, Spain.

Cecilia Martinez. Hospital Clínico Universitario de Valencia. Valencia, Spain.

Elena Balmaseda. Complejo Hospitalario Universitario de Albacete. Albacete, Spain.

Javier Rubio. Hospital Universitario de Jerez de la Frontera. Jerez de la Frontera, Spain.

Enrique La Orden. Hospital Universitario Infanta Elena. Valdemoro, Spain.

Carolina Gutierrez. Hospital Universitario Puerta de Hierro. Majadahonda, Spain.

Helena Lorenzo. Hospital Universitario de Basurto. Bilbao, Spain.

Sonia Fernandez. Hospital Universitario Severo Ochoa. Leganés, Spain

Cristina Molinos. Hospital Universitario de Cabueñes. Gijón, Spain.

Beatriz Espin. Hospital Universitario Infantil Virgen del Rocío. Sevilla, Spain.

Mª del Carmen Miranda. Hospital Universitario Infanta Cristina. Parla, Spain.

Ricardo Torres. Hospital Universitario de Salamanca. Salamanca, Spain.

Raquel Vecino. Hospital Clínico San Carlos. Madrid, Spain.

Mercedes Juste. Hospital Universitario San Juan de Alicante. Alicante, Spain.

Carmen Alonso. Hospital Clínico Universitario de Valladolid. Valladolid, Spain.

Miriam Blanco. Fundación Jimenez Díaz. Madrid. Madrid, Spain.

Begoña Perez-Moneo. Hospital Infanta Leonor. Madrid, Spain.

Amadeu Roca. Hospital Comarcal Sant Jaume de Calella. Calella, Spain.

Roger García. Hospital Mutua de Terrassa. Terrassa, Spain.

Jose Manuel Marugan. Hospital Clínico Universitario de Valladolid. Valladolid, Spain

Antonio Grande. Hospital Universitario de Salamanca. Salamanca, Spain.

Margarita Pich. Centro Medico Teknon. Barcelona, Spain.

Marta Soria. Hospital HM Montepríncipe/Torrelodones. Torrelodones, Spain.

Maria de las Mercedes Busto. Complejo Hospitalario de Pontevedra. Pontevedra, Spain

Santiago Fernandez Cebrián. Complejo Hospitalario Universitario de Ourense. Ourense, Spain.

Luis Grande. Hospital Universitario de Getafe. Getafe, Spain.

Elena Crehuá. Hospital Clínico Universitario de Valencia. Valencia, Spain.

Mercedes Sebastián. Hospital Universitario de Móstoles. Móstoles, Spain.

Jose Vicente Arcos. Hospital Sant Joan de Deu. Barcelona. Barcelona, Spain.

Idoia Hualde. Hospital Universitario de Araba – Txagorritxu. Vitoria, Spain.

Ariane Calvo. Hospital Universitario de Araba – Txagorritxu, Spain.

Andres Bodas. Hospital Clínico San Carlos. Madrid, Spain.

Rosa Ana Muñoz. Hospital Infantil Universitario Niño Jesús. Madrid, Spain.

David Gil. Hospital Universitario Virgen de la Arrixaca. Murcia, Spain.

Eduardo Ubalde. Hospital General San Jorge. Huesca. Huesca, spain.

Ana Arévalo. Centro Hospitalario Ciudad de Jaén. Jaén, Spain.

Maria Aurora Pérez. Centro Hospitalario Ciudad de Jaén. Jaén, Spain.

Gonzalo Botija. Hospital Universitario Fundación Alcorcón. Alcorcón, Spain.

Juan Pablo Ferrer. Hospital General de Requena. Requena, Spain.

Ana Isabel Ruiz. Hospital El Escorial. San Lorenzo del Escorial, Spain.

Mª Angeles Gómez. Hospital Universitario Virgen del Castillo. Yecla, Spain.

Victor Manuel Navas. Hospital Materno Infantil de Malaga. Málaga, Spain.

Encarnación Maria Lancho. Hospital Universitario del Tajo. Aranjuez, Spain.

Rafael Gonzalez de Caldas. Hospital Universitario Reina Sofía. Córdoba, Spain.

Juan Manuel Bartolomé. Complejo Asistencial Universitario de Palencia. Palencia, Spain.

Pablo Oliver. Hospital de Mendaro. Mendaro, Spain.

Mª Jesus Balboa. Hospital Universitario Juan Ramón Jiménez. Huelva, Spain.

Lissette Delgado. Hospital Alto Deba: Mondragón, Spain.

Carlos Trillo. Hospital de La Axarquía. Vélez, Spain.

José Bernardo González. Hospital Universitario de Burgos. Burgos, Spain.

Francisco Perez. Hospital Infanta Margarita. Cabra, Spain.

Guillermo Mascort. Hospital Infanta Margarita. Córdoba. Spain.

Jose Ignacio Olazábal. Centro de Salud La Calzada II. Gijón, Spain.

Haydeé Expósito. Hospital Nuestra Señora de Sonsoles. Avila, Spain.

Juana Mª Rizo. Hospital Universitario Infanta Sofia. San Sebastián de los Reyes, Spain.

Margarita Revenga. Hospital Universitario La Zarzuela. Madrid, Spain.

Laura Alonso. Hospital Universitario HM Sanchinarro. Madrid, Spain

Miguel Angel Carro. Hospital Universitario General de Villalba. Villalba, Spain

Elisenda Busquets. Hospital Universitario Parc Taulí. Sabadell, Spain

Myriam Herrero. Hospital Universitario Rey Juan Carlos. Móstoles, Spain
